# Effects of maize organ-specific drought stress response on yields from transcriptome analysis

**DOI:** 10.1186/s12870-019-1941-5

**Published:** 2019-08-01

**Authors:** Baomei Wang, Can Liu, Dengfeng Zhang, Chunmei He, Juren Zhang, Zhaoxia Li

**Affiliations:** 1 Key Laboratory of Plant Cell Engineering and Germplasm Innovation, School of Life Sciences, Shandong University, Qingdao, 266237 Shandong China; 2 Institute of Crop Sciences, Chinese Academy of Agricultural Sciences, Beijing, 100081 China; 3 Maize Research Institute, Shandong Academy of Agricultural Sciences, Jinan, 250100 Shandong China

**Keywords:** Drought, Maize, Yield, Comparative transcriptomics analysis, Ear, Ear leaf, Kernel

## Abstract

**Background:**

Drought is a serious causal factor of reduced crop yields than any other abiotic stresses. As one of the most widely distributed crops, maize plants frequently suffer from drought stress, which causes great losses in the final kernel yield. Drought stress response in plants showed tissue- and developmental stage-specific characteristics.

**Results:**

In this study, the ears at the V9 stage, kernels and ear leaf at the 5DAP (days after pollination) stage of maize were used for morphological, physiological and comparative transcriptomics analysis to understand the different features of “sink” or “source” organs and the effects on kernel yield under drought stress conditions. The ABA-, NAC-mediate signaling pathway, osmotic protective substance synthesis and protein folding response were identified as common drought stress response in the three organs. Tissue-specific drought stress responses and the regulators were identified, they were highly correlated with growth, physiological adaptation and yield loss under drought stress. For ears, drought stress inhibited ear elongation, led to the abnormal differentiation of the paired spikelet, and auxin signaling involved in the regulation of cell division and growth and primordium development changes. In the kernels, reduced kernel size caused by drought stress was observed, and the obvious differences of auxin, BR and cytokine signaling transduction appeared, which indicated the modification in carbohydrate metabolism, cell differentiation and growth retardation. For the ear leaf, dramatically and synergistically reduced the expression of photosynthesis genes were observed when suffered from drought stress, the ABA- and NAC- mediate signaling pathway played important roles in the regulation of photosynthesis.

**Conclusions:**

Transcriptomic changes caused by drought were highly correlated with developmental and physiological adaptation, which was closely related to the final yield of maize, and a sketch of tissue- and developmental stage-specific responses to drought stress in maize was drafted.

**Electronic supplementary material:**

The online version of this article (10.1186/s12870-019-1941-5) contains supplementary material, which is available to authorized users.

## Background

Due to anchoring by the root to a fixed spot, plants encounter various environmental stresses that affect growth and development during their life cycle. Among the environmental stresses, drought is one of the most serious stresses and ultimately causes crop yield reductions [1]. Maize is one of the most widely distributed food crops worldwide, with a total production surpassing that of wheat or rice [2]. Maize yield is severely limited by water [3].

The growth stages of maize can be divided into seedling growth (VE and V1), vegetative growth (V2, V3... Vn), flowering and fertilization (VT, R0, and R1) and grain filling and maturity (R2 to R6) [4]. Drought stress during the vegetative period can cause a reduced growth rate, prolong the vegetative growth stage, redirect the roots and alter the carbohydrate distribution in maize. Short-duration water deficits have been shown to cause 28–32% losses of dry weight during the rapid vegetative growth stage and 66–93% losses of dry weight during the tasseling and ear formation stages, respectively [5]. At the V9 stage, many potential ear shoots arise, and the number of kernel rows is determined [4, 6]. At approximately the V10 stage, the corn plant begins a rapid, steady increase in nutrient and dry weight accumulation, which continues into the reproductive stage. Additionally, long-term drought (21 days) during the pre-flowering stage has been shown to reduce the final sizes of certain leaves and internodes, delay tassel and silk emergence, and cause yield losses from 15 to 25% [7]. The potential of kernels per row is determined by at least V15 and potentially as early as the V12 stage, as drought stress at this stage will dramatically reduce the kernel number per plant [8, 9]. Five days of drought stress around the pollination stage results in abnormal embryo formation and a markedly decreased kernel number [10]. A decreased kernel set, primarily in apical ear regions, has also been observed in response to 5 days of drought stress during the pre-pollination and early post-pollination stages [11]. The ear leaf of maize contributes greatly to the accumulation of biomass due to photosynthesis. The photosynthate for kernel yield is largely produced by five or six leaves near and above the ear [12, 13]. Drought stress results in a dramatic decline in the photosynthetic rate, which hampers growth by reducing the size of the “source” in plants [9].

Gene regulation networks of drought stress response were surveyed and summarized mostly by using plants at the seedling stage. Numerous genes were identified during drought and osmotic stress in diverse plants (reviewed in [14–18]). Generally, they can be divided into abscisic acid (ABA)-dependent and other signaling pathways [19]. ABA is a major phytohormone involved in the drought stress response in plants; it is involved in stomatal closure and stress-responsive gene expression [19–21]. Other signaling pathways for drought stress include osmotic stress signaling [22, 23], the calcium-dependent pathway [24, 25], mitogen-activated kinase-mediated signaling [26, 27], phospholipid signaling [28–30] and reactive oxygen species (ROS) signaling [31, 32], among others.

Although global gene expression profiles in response to water deficit have been monitored in different tissues of maize by microarray hybridization and RNA-sequence experiments [33–40], the co-expression networks of different organs have not been established, and the correlation their contribution to the final yield have not yet been conducted. Plant responses to stress are dependent on the affected tissue or organ that performs different functions. The level and duration of stress (acute vs chronic) can have a significant effect on the complexity of the response [41]. In plants, the coordination of organ-specific response and whole-plant response to drought stress, the balance of tolerance and growth need to considered. Is the developmental retardation caused by drought stress consistent with the reduction of corn grain yield? What is the mechanism of yield reduction? It will be interesting to answer these questions.

**Fig. 1:**
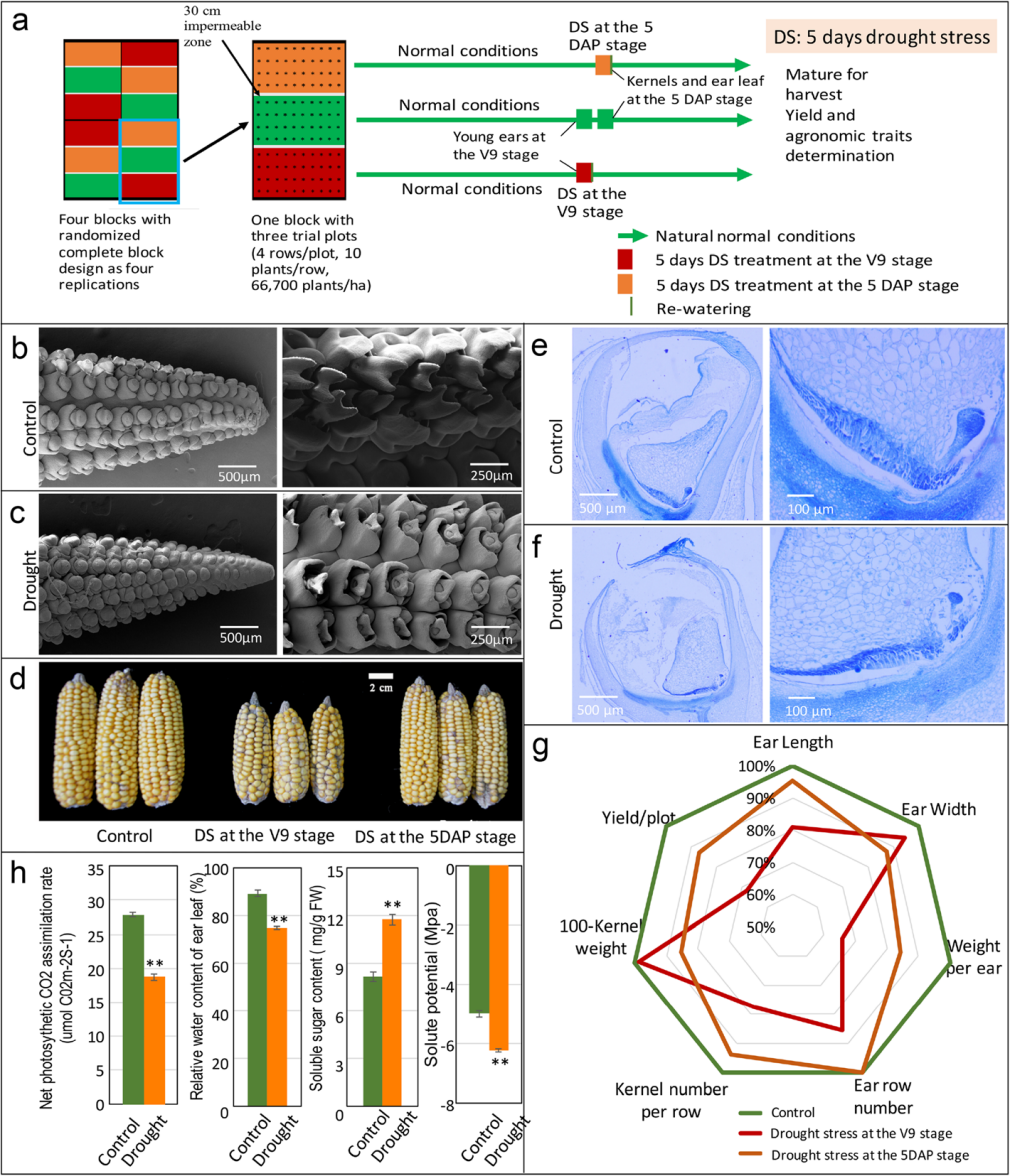
Experiment design, ear development, biomass accumulation in the kernel, agronomic traits changes in the plants and physiological changes in the ear leaf response to drought stress. **a** Schematic diagram of the experiment design, the stage of drought stress treatment and samples collection. **b** and **c** Morphological characteristics of the ears at the V9 stage under normal conditions (**b**) and drought conditions (**c**). **d** Mature ears from plants under control and drought conditions. **e** and **f** Morphological characteristics of the kernels and the basal endosperm transfer cell layer (BETL) at the 5DAP stage under normal conditions (**e**) and drought conditions (**f**). **g** Radar chart of agronomic traits changes of maize plants grew under control and drought stress conditions (at the V9 stage and 5 DAP stage). **h** Net photosynthesis, relative water content, soluble sugar content, and solute potential of the ear leaf at the 5DAP stage under control and drought conditions. All the plants were grown under natural conditions, normal nutrients, well-watered soil in the field until the designated stages. Two different drought stress treatments (5 days drought stress at the V9 stage (when the 10th leaf appeared) and 5DAP stage (when the silk appeared)) and a normal control were performed. The upper ear (at the V9 stage), the ear leaf and kernels on the upper ear (at the 5 DAP stage) from drought stress and control plants was collected for morphological analysis. After re-watering, the remaining plants were grown to maturity under suitable conditions, and then the agronomic traits were determined (original data in Additional file 1: Table S1). At least three biological repeats were sampled for a treatment, and each repeat contained organs from 4 plants. Values are the means of the replicates ± sd. ** was statistical significance with *P* < 0.01 by using a t-test

In this study, when subjected to drought stress, three maize organs with different biological characteristics, including young ears on plants at the V9 stage and ear leaf and kernels of plants at 5 days after pollination (DAP), showed high correlations to the final yield of maize. To understand the coordination of the organ-specific and whole-plant drought stress response, the balance of “sink” (ear and kernel) and “source” (era leaf), a comparative transcriptome analysis was performed using the three organs mentioned from plants with or without drought stress treatment (Fig. 1a). Metabolism adjustment, osmotic adjustment, and protein folding acclimation were observed in all organs after drought treatment, and ABA-dependent and NAC-mediated stress response pathways were found. For the ear leaf, genes encoding photosynthesis proteins and net photosynthesis were dramatically and synergistically reduced, and the ABA- and NAC- mediate signaling pathway played important roles in the transcriptional regulation. For “sinks”, cell division, cell growth and primordium development were delayed in the ear, and endosperm differentiation was delayed in 5DAP kernel. Based on the transcriptome analysis, phytohormone signaling was involved in the subtle control and regulation process, especially auxin. A hypothesis integrating the metabolism adjustment, signaling alterations, and stress-protective processes is presented.

## Results

### Drought stress decreased the net photosynthesis rate and caused abnormal development

As elucidated in maize development, the ear at the V9 stage was undergoing primordium development, with the primordia determining the kernel rows. Kernel rows first initiate as “ridges” of cells that eventually differentiate into pairs of rows [4, 6]. Many potential spikelets were developing, and the number of kernel rows was determined at this stage. As shown in Additional file 1: Figure S1A, the average length of the ear was 2.55 cm under normal conditions and 1.95 cm when subjected to 5 days of drought stress. When the subsequent primordia were observed by scanning electron microscopy (SEM), their growth and development were delayed in plants subjected to drought stress, especially the upper part of the ear. For ears under control conditions, all the following primordia grow well to the tip of the ear, while in the ears subjected to drought, the development of subsequent primordia on the top part failed (Fig. 1b and c). As reflected in the mature ear (Fig. 1d and Additional file 1: Table S1), the ear length under drought stress conditions was significantly shorter than in the control (10.01 cm for drought stress and 12.36 cm for the control, grain numbers per row of 17.33 and 22.33, respectively), and the row number of ears was 12 under drought and 14 in the normal control. This showed that drought stress at the V9-V10 stage caused a reduced row number and no kernels on the upper part of the mature ear due to the disturbance of primordium development by drought stress.

Another stage that is sensitive to drought stress in maize is the grain fill period after pollination. During DAP1–4, the endosperm is composed of a large coenocyte in which nuclear divisions occur without cellularization [42, 43]. At approximately 6DAP, the endosperm differentiates into starchy endosperm, the basal endosperm transfer layer, the aleurone layer, and the embryo-surrounding region [43–46]. Five days drought stress treatment was performed after pollination for 24 h, and the kernels after the treatment were collected for morphological analysis. Paraffin sections indicated that whole kernel development was delayed, and the development of the basal endosperm transfer layer and embryo was particularly dramatically arrested by drought stress (Fig. 1e and f). Following maturation, the 100-grain dry weight was 20.33 g and 17.38 g, and kernel weight per ear was 59.75 g and 50.38 g under normal and drought stress conditions, respectively (Fig. 1g and Additional file 1: Table S1). As shown in Fig. 1g, there was difference in row number and slightly shorter ear length, but smaller kernels were observed between the plants subjected to drought stress and the control. The reduced grain weight by drought at this stage was due to the decrease in kernel size.

The ear leaf contributes greatly to the “source” for ear and kernel development as a photosynthesis factory. Here, the performance of the ear leaf at the 5DAP stage with and without drought stress treatment was observed. As shown in Fig. 1h, drought stress treatment led to a significant decline of the net photosynthetic rate (27.87 to 18.73 μmol CO_2_m^−2^S^− 1^, normal control to drought stress) and relative water content (RWC, 89 to 74%), while the soluble sugar contents (8.18 to 11.74 mg/g) and solute potential (− 6.34 Mpa to − 5.00 Mpa) were significantly increased. The analysis showed that the capability for photosynthesis was significantly arrested by drought stress treatment, and the “source” was reduced. Ultimately, the yield per plot was 2.31 kg under normal conditions and 1.57 kg (drought at V9) or 2.01 kg (drought at 5DAP) under drought stress (Fig. 1g and Additional file 1: Table S1). The yield loss during the treatment was due to the reduced “source” (soluble sugar content in leaves) and the “sink” (young ear or kernels).

### Overall differentially expressed genes (DEGs) in three organs in response to drought stress

**Fig. 2:**
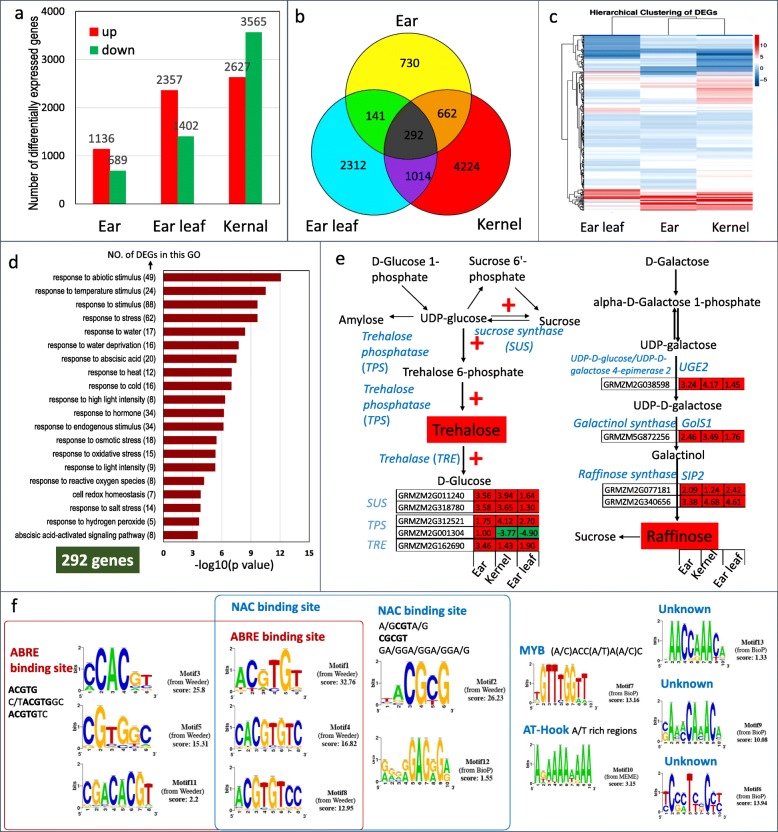
Overall differentially expressed genes (DEGs) in response to drought stress in ears at the V9 stage and in the ear leaf and kernel at the 5DAP stage. **a** The number of DEGs in ears at the V9 stage and the ear leaf and kernels at 5DAP. **b** Venn diagram showing the overlap of DEGs in the ear at the V9 stage and the ear leaf and kernels at 5DAP. **c** Heat map of the DEGs in the ear at the V9 stage and in the ear leaf and kernels at 5DAP in response to drought stress. **d** GO analysis of the 292 DEGs in all three organs. **e** DEGs in the synthesis of sucrose, trehalose and raffinose. **f** Upstream sequence motifs analysis of the 292 DEGs in all three organs. The upstream regions of the genes for common sequence motifs using PromZea (http://128.196.172.219/index.html). All the plants were grown under natural conditions, normal nutrients, well-watered soil in the field until the designated stages. Two different drought stress treatments (5 days drought stress at the V9 stage and 5DAP stage) and a normal control were performed. The upper ear (at the V9 stage), the ear leaf and kernel on the upper ear (at the 5 DAP stage) from drought stress and control plants was collected for RNA sequencing. Two biological repeats were sampled for a treatment, and each repeat contained organs from 4 plants for one library. The absolute values of log2 (drought/control) ≥1 and FDR < 0.001 were used as the criteria for DEGs. The color of the box represents up (red) and down (green)-regulated genes (drought/normal), and the value in the box is the log2 (drought/control) of the genes in the corresponding organs

Young ear at the V9 stage and ear leaf and kernel at 5DAP from plants subjected to drought stress and grown under control conditions were used for RNA sequencing to identify the DEGs and pathways in response to drought stress (GEO Submission (GSE132113)). The total reads, base pairs, mapped reads, expressed genes and transcripts were listed in Additional file 1: Table S2. More expressed transcripts were identified in the ear and kernel compared to ear leaf. The latter consisted of differentiated and terminal tissues. As summarized in Fig. 2a, compared with the controls, 1136 upregulated and 689 downregulated genes were observed in the ear, 2357 upregulated and 1402 downregulated genes in the ear leaf, and 2627 upregulated and 3565 downregulated genes in the kernel. When comparing the DEGs in different tissues, 292 genes showed consistent changes in the three tissues. The kernel shared 662 DEGs with the ear and 1014 DEGs with the ear leaf, while only 141 DEGs were shared in the ear and ear leaf (Fig. 2b and c). To confirm the results, 22 genes with different transcript abundances were validated by real-time RT-PCR (Additional file 1: Figure S2). The expression of these genes showed good consistency between the two detection methods.

Gene Ontology (GO) terms for response to light intensity, response to stress (drought, heat and oxidative stress), and regulation of the ABA-mediated signaling pathway were significantly enriched (Fig. 2d). Common drought response pathways identified in the three organs included the ABA-dependent signaling pathway, osmotic protective substance synthesis, protein folding and NAC TF (transcription factor)-mediated drought response (Fig. 2e, Additional file 2: Table S3). In all three organs, genes encoding the enzymes involved in the synthesis of sucrose, trehalose, raffinose and proline and genes encoding peroxidase, thioredoxin and oxidative stress, which contribute to ROS scavenging, were also largely induced by drought stress (Additional file 2: Table S3). Late embryogenesis abundant and some other drought-induced protein families were also accumulated (Additional file 2: Table S3), which could facilitate the adaptation of plants to water deficit. The other significantly changed process was the protein folding process, including the dramatic induction of heat shock protein (HSP). As shown in Additional file 2: Table S3, genes encoding HSP 101, HSP 70 and small HSPs were dramatically induced by drought stress, especially the small HSPs (five HSP20, two HSP17.6, and one DNAJ-like 20). Two heat shock TFs were also largely induced: one was the homolog of AtHSFB2A (GRMZM2G098696, 8.11-fold (log 2 value was 3.02, both log 2 value and ratio in the supplemental tables and log 2 value in the figures) in the ear, 5.78-fold in the ear leaf and 3.81-fold in the kernel), and the other was the homolog of AtHSFC1 (GRMZM2G105348, 81.91-fold in the ear, 2.73-fold in the ear leaf and 97.21-fold in the kernel). AtHSFB2A was a key component of heat stress signaling [47, 48]. These heat shock TFs may act as a connection between drought stress and cytosolic protein folding. Metabolic adjustment, osmotic adjustment, and protein folding acclimation were observed in all organs after drought treatment, and therefore, we deduced that a common mechanism regulates these responses.

To better understand the coordinate regulation of genes in all the tissues, we analyzed the upstream regions of the 292 genes for common sequence motifs using PromZea. Thirteen candidate motifs were identified enriched in the promoters of these genes (Fig. 2f), 6 of them with the core ABA-responsive element (ABRE) binding site (ACGTG) and 5 with identified NAC TFs binding site. That means two candidate regulatory systems were played roles in all the tissues: ABA-dependent and NAC-mediated stress response pathways. As shown in Additional file 2: Table S3, the ABA biosynthesis genes β-Ohase 1 (GRMZM2G382534, 4.84-, 2.49- and 42.40-fold in the ear, kernel and ear leaf respectively) and NCED9 (GRMZM2G014392, 4.33-, 4.28-, and 3.57-fold in the ear, kernel and ear leaf respectively) were induced by drought stress. Interestingly, 12 ABA-induced PP2Cs were also significantly upregulated by drought stress, including four HAI (Highly ABA-Induced) homologs and eight HAB (Hypersensitive to ABA) homologs, which work as positive and negative regulator of ABA signaling [49, 50]. Twenty TFs were identified as differentially expressed in all three organs. Five NAC TFs were significantly induced, including three ATAF2 homologs (GRMZM2G347043, 5.95-, 35.21- and 8.39-fold upregulated, GRMZM2G336533, 30.40-, 17.16- and 13.36-fold upregulated and GRMZM2G123667, 10.46-, 2.50- and 3.61-fold upregulated in the ear, kernel and ear leaf respectively), one NAC002/ATAF1 homolog (GRMZM2G014653, 5.97-, 16.56 and 3.13-fold upregulated in the ear, kernel and ear leaf) and one NAC047 (GRMZM2G134073, 2.23- and 2.33-fold upregulated in the kernel and ear leaf). All these NAC TFs have been reported to be involved in the stress response and development [51, 52]. Overexpression of NAC002/ATAF1 activates TREHALASE1 expression and leads to reduced trehalose-6-phosphate levels and a reduced sugar starvation metabolome [53]. In addition, ATAF2 regulates auxin biosynthesis [54] and BR catabolism [55], while NAC002/ATAF1 regulates ABA biosynthesis [56] in Arabidopsis. The upregulation of ATAF1/2 homolog genes in the maize response to drought stress may not only be involved in the sugar starvation response but also impact plant hormone levels and metabolic adjustment.

### Effects of drought stress on young ear development and cell division based on transcriptome analysis

**Fig. 3:**
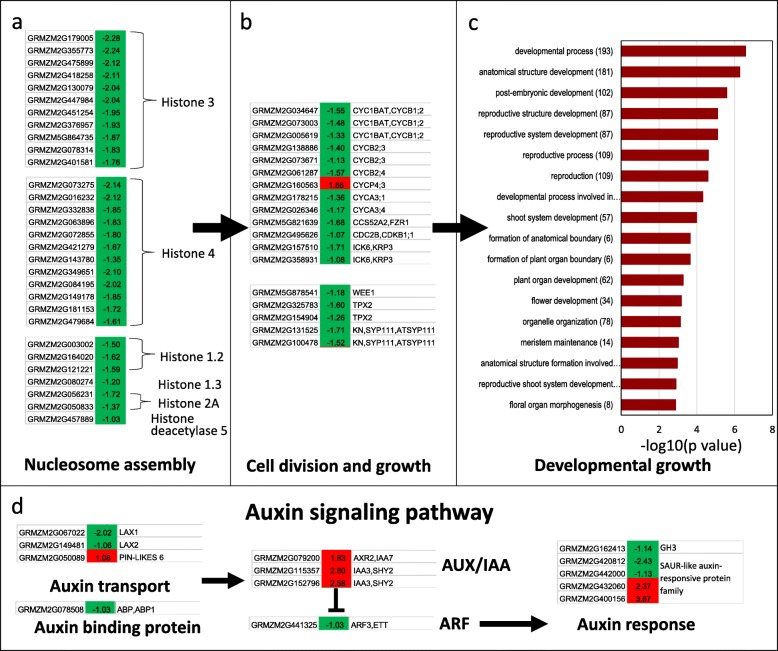
DEGs in ears at the V9 stage in response to drought were involved in nucleosome assembly, cell division and growth, auxin signaling and the development process. **a** Downregulated genes involved in nucleosome assembly. **b** Downregulated genes involved in cell division and growth. **c** GO term analysis of DEGs in ears at V9 stage showed enrichment of the organ development process. **d** DEGs involved in the auxin signaling pathway. All the plants were grown under natural conditions, normal nutrients, well-watered soil in the field until the designated stages. Drought stress treatments (5 days drought stress at the V9 stage) and a normal control were performed. The upper ear from drought stress and control plants was collected for RNA sequencing. The absolute values of log2 (drought/control) ≥1 and FDR < 0.001 were used as the criteria for DEGs. The color of the box represents up (red) and down (green)-regulated genes (drought/normal), and the value in the box is the log2 (drought/control) of the genes in the ear (drought/normal) at the V9 stage

As shown in Fig. 1, drought stress at the V9 stage significantly inhibited primordium development, which contributed to the spikelets and determined the number of kernels and the ear weight. Transcriptome analysis showed that the expression of genes involved in nucleosome assembly, cell division and growth for the formation of anatomical boundaries were significantly altered by drought stress (Fig. 3, Additional file 2: Table S4). Eleven genes encoding histone 3, 12 genes encoding histone 4, 2 genes encoding histone 2, and 4 genes encoding histone 1 were significantly downregulated by drought stress (Fig. 3a). The downregulation of CYC1; 2, CYC2; 3, CYC3; 1 and CYC3; 4 indicated a reduction of cell division (Fig. 3b, Additional file 2: Table S4). Identification of the regulator in this process is merited to modify the maize yield under drought stress conditions. By comparison, auxin was the most altered hormone signaling cascade, as demonstrated by the inhibited expression of two AUX1-like auxin influx transporters (GRMZM2G067022, 0.25-fold and GRMZM2G149481, 0.48-fold), the downregulation of ABP1 (auxin-binding protein 1, GRMZM2G078508, 0.49-fold), and the upregulated expression of one auxin efflux transporter (GRMZM2G050089, 2.12-fold), one IAA7/AXR2 (GRMZM2G079200, 3.55-fold), two IAA3/SHY2 (GRMZM2G115357, 6.97-fold and GRMZM2G152796, 5.99-fold) and some other auxin response genes such as GH3 and SAUR-like auxin-responsive family genes (Fig. 3d). Both the *axr2–1* and *axr3–1* exhibited strong insensitivity to ABA for embryonic axis elongation [57–59] . IAA3/SHY2, AUX1/LAX3 are required for auxin signaling that activates LBD16/ASL18 and LBD18/ASL20 to control lateral root development [60, 61]. Analysis of the DEGs in auxin signaling pathways revealed that these IAA-modulated developmental processes were altered in drought stress, which induced growth retardation. The GO terms relative to development and growth were also significantly different between control and drought conditions (Fig. 3c). The decreased cell division and growth due to drought stress caused a change in meristem activity and led to modified structural development.

Here, the significant upregulation of four NAC081/ATAF2 (GRMZM2G347043, 5.95-fold, GRMZM2G123667, 10.36-fold, GRMZM2G068973, 14.56-fold and GRMZM2G336533, 30.40-fold) and one NAC002/ATAF1 (GRMZM2G014653, 5.97-fold) and the downregulation of the homologs of NAC031/CUC3 (GRMZM2G430522, 0.45-fold), NAC098/CUC2 (GRMZM2G139700, 0.42-fold) and NAC047 (GRMZM2G134073, 0.40-fold) in response to drought stress treatment were observed compared with the ears under normal conditions (Additional file 2: Table S4). CUC2 and CUC3 have been reported to participate in the regulation of shoot meristem boundary and formation and subsequent development [62, 63]. ATAF1/2 have been reported not only as the central regulator of plant defense and light-mediated seedling development but also as a regulator of hormone metabolism, such as BR, auxin and ABA [54–56]. The downregulation of homologs of NAC031/CUC3, NAC098/CUC2 and NAC047 in response to drought stress treatment undoubtedly retarded ear development. The collaborative regulation of auxin, ABA and NAC was involved in the responses to drought stress in ear development in maize.

### Effects of drought stress on kernel development and yield

**Fig. 4:**
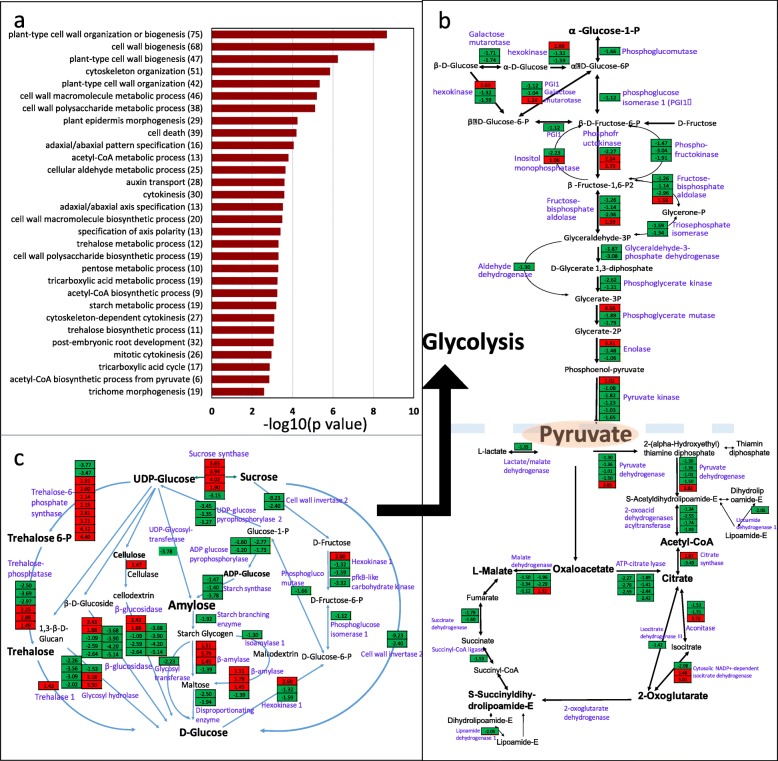
Go terms and pathway analysis of DEGs in kernels at the 5DAP stage showed that carbohydrate metabolism and development process were significantly changed after drought stress. **a** GO term analysis of the DEGs in kernels in response to drought stress showed an enrichment in carbohydrate metabolism and organ development process. **b** DEGs in carbohydrate metabolism (glycolysis and TCA cycle). **c** DEGs in carbohydrate metabolism (Starch and sucrose metabolism). All the plants were grown under natural conditions, normal nutrients, well-watered soil in the field until the designated stages. Drought stress treatments (5 days drought stress at the 5DAP stage) and a normal control were performed. The kernel on the upper ear from drought stress and control plants was collected for RNA sequencing. The absolute values of log2 (drought/control) ≥1 and FDR < 0.001 were used as the criteria for DEGs. The color of the box represents up (red) and down (green)-regulated genes (drought/normal), and the value in the box is the log2 (drought/control) of the genes in the kernel at the 5DAP stage. Schematic of the pathways modified according to the KEGG

As shown in Fig. 1, the significant developmental change caused by drought at the 5DAP stage was a delay or abnormal endosperm differentiation (Fig. 1), which ultimately led to a small kernel size. GO terms related to metabolic process, cell wall biosynthesis process, cytokinesis, epidermal morphogenesis and organ development were enriched in response to drought stress at this stage (Fig. 4a, Additional file 2: Table S5). A significant change was observed in carbohydrate metabolism pathways. Glycolysis and TCA pathway activities were significantly reduced; however, the synthesis of trehalose, which aids in the osmotic adjustment response to stress, was significantly increased (Fig. 4b). Amylose synthesis was decelerated (Fig. 4c), and disaccharide and polysaccharide utilization was accelerated, potentially due to the reduced supply of photosynthate. Moreover, under unfavorable conditions, the kernel attempted to use more carbohydrates to cope with the unfavorable environment instead of undergoing rapid cell divisions and cellularization.

**Fig. 5:**
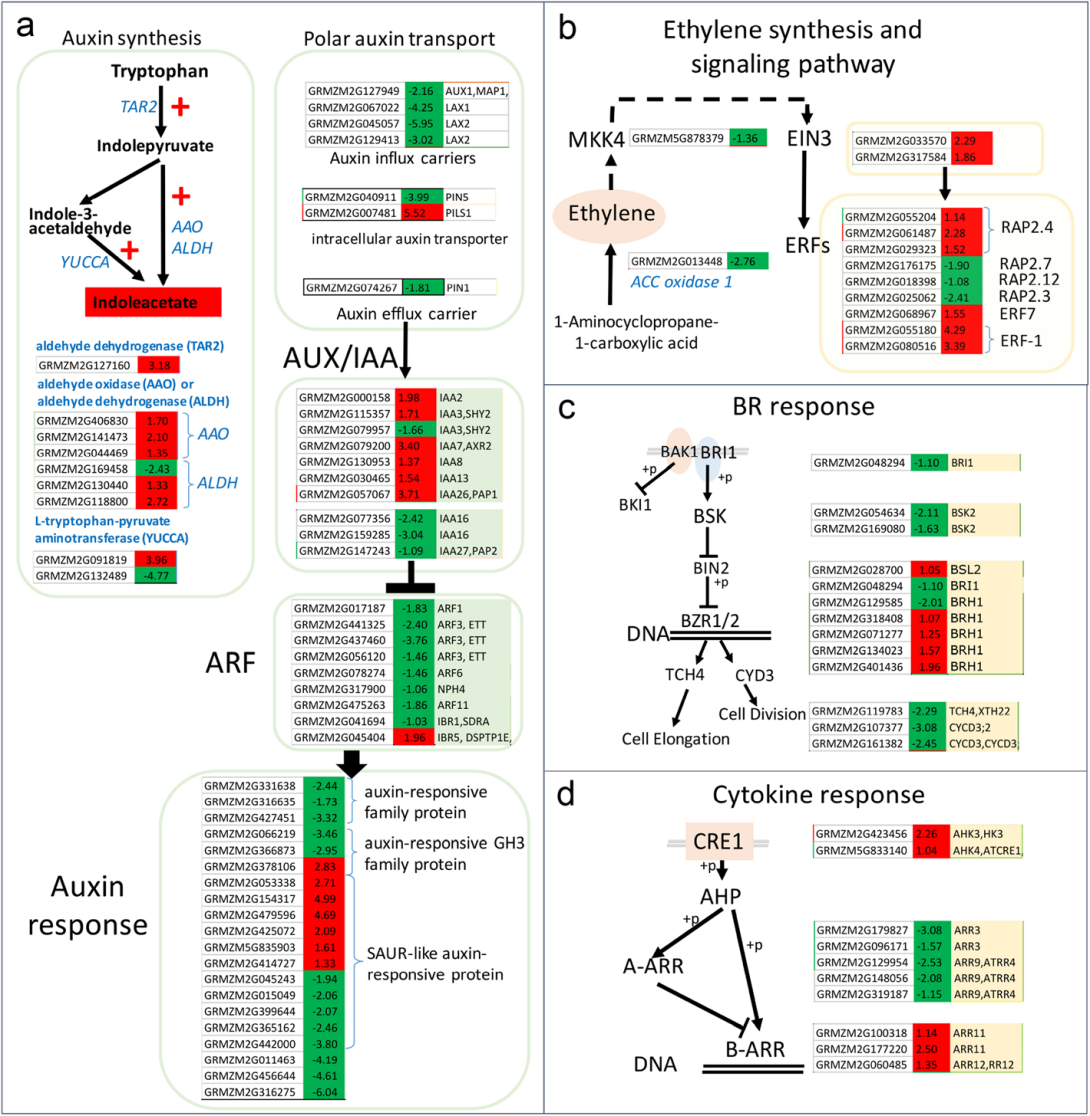
DEGs in kernels at the 5DAP stage involved in plant hormone signaling pathways. **a** DEGs involved in the auxin signaling pathway. **b** DEGs involved in the ethylene signaling pathway. **c** DEGs involved in the BR signaling pathway. **d** DEGs involved in the cytokine signaling pathway. All the plants were grown and treated as described in Fig. 4. The kernel on the upper ear from drought stress and control plants was collected for RNA sequencing. The absolute values of log2 (drought/control) ≥1 and FDR < 0.001 were used as the criteria for DEGs. The color of the box represents up (red) and down (green)-regulated genes (drought/normal), and the value in the box is the log2 (drought/control) of the genes in the kernel at the 5DAP stage. Schematic of the pathways modified according to the KEGG

During kernel development, phytohormones play important roles, especially auxin signaling. As shown in Fig. 5 and Additional file 2: Table S5, auxin polar transport was reduced by drought stress because of the downregulation of four AUX1/LAX genes (AUX1/GRMZM2G127949, 0.22-fold, LAX1/GRMZM2G067022, 0.05-fold, LAX1/GRMZM2G045057, 0.02-fold, and LAX2/GRMZM2G045057, 0.12-fold) and one PIN1 gene. ZmPIN1-mediated auxin fluxes facilitate auxin accumulation and promote kernel differentiation [64]. In this study, both auxin influx and efflux were affected by drought stress. Local auxin biosynthesis was promoted, especially the conversion of tryptophan to indole-3-acetaldehyde and then to indoleacetate (IAA) (Fig. 5a), and the input was reduced. Together with the change in the local auxin concentration, auxin signaling was also altered. As shown in Fig. 5a and Additional file 2: Table S5, IAA2 (3.95-fold), IAA3 (3.28-fold), IAA7 (10.52-fold), IAA8 (2.58-fold), IAA13 (2.90-fold) and IAA26 (13.06-fold) were upregulated and two IAA16 (0.19-fold and 0.12-fold), one IAA27 (0.47-fold) and one IAA3 (0.32-fold) were downregulated in the kernel in response to drought stress treatment compared with the control. Accompanying AUX/IAA, one ARF1 (0.28-fold), three ARF3 (0.19-, 0.36- and 0.07-fold), one ARF6 (0.36-fold), one ARF11 (0.28-fold) and an IBR1 (0.49-fold) were downregulated. The degradation of Aux/IAA proteins could free ARFs, and the latter could activate or repress auxin response gene expression directly. Auxin response genes such as SAUR and GH3 were differentially expressed compared with the control in the kernel under drought stress conditions. The differential expression of genes involved in auxin transport, local biosynthesis and signaling pathway may modify metabolism to establish a new hemostasis in kernels subjected to drought stress at this stage.

In addition to auxin, ethylene, BR and cytokine signaling were also changed to cope with the stress environment and to adjust growth or development. As shown in Fig. 5b, two EIN3 genes were upregulated (GRMZM2G033570, 4.88-fold and GRMZM2G317584, 3.62-fold) and their downstream RAP/ERF TFs showed varied expression levels. RAP/ERF TFs can help with the stress tolerance of plants. Compared with ethylene, BR and cytokines seem to function in growth and developmental regulation to acclimate to drought stress (Fig. 5c-d). Genes encoding BRI1 (GRMZM2G048294, 0.47-fold downregulated) and BSK2 (GRMZM2G169080, 0.32-fold and GRMZM2G054634, 0.23-fold downregulated), which were considered as main target of BR to regulate plant growth were differentially expressed. Their targets TCH4 (GRMZM2G119783, 0.20-fold), which contributes to cell elongation, and two CYCD3 (GRMZM2G107377, 0.12-fold and GRMZM2G161382, 0.18-fold), which contribute to cell division, were inhibited. Interestingly for cytokine signaling, five A-ARR were identified as downregulated (GRMZM2G096171, 0.34-fold, GRMZM2G179827, 0.12-fold, GRMZM2G129954, 0.17-fold, GRMZM2G319187, 0.45-fold, GRMZM2G148056, 0.24-fold), and three B-ARR were upregulated (GRMZM2G177220, 5.66-fold, GRMZM2G100318, 2.20-fold and GRMZM2G060485, 2.55-fold). B-type ARRs, ARR1, ARR10, and ARR12 redundantly act as negative regulators of drought responses in both ABA-dependent and -independent pathways [65] and mediate cytokinin signaling to WUS for the maintenance of stem cells [66]. Based on these findings, plant hormones play important roles in the adaptation of metabolism in kernels subjected to drought stress, including reduced cell division, expansion, differentiation and embryo development at this stage.

### Effects of drought stress on ear leaf transcriptome and photosynthesis

**Fig. 6:**
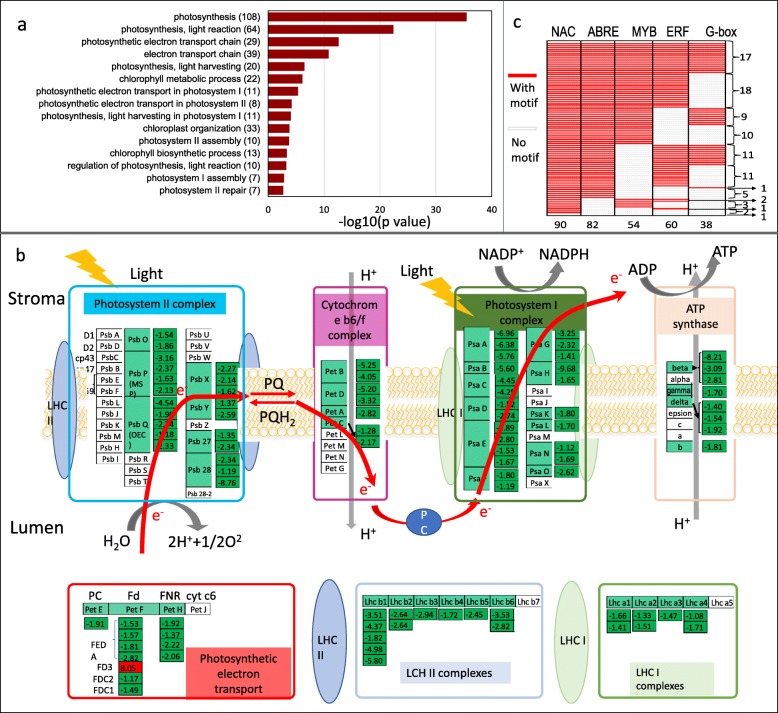
DEGs in the ear leaf (drought/normal) at the 5DAP stage showed reduced expression of genes involved in photosynthesis. **a** GO term analysis of the DEGs in the ear leaf (drought/normal) at the 5DAP stage. **b** Downregulated genes involved in photosynthesis and photosynthesis antenna proteins. **c** Upstream sequence motifs analysis of the DEGs (coded by nuclear DNA) involved in photosynthesis and photosynthesis antenna proteins. All the plants were grown and treated as described in Fig. 4. The ear leaf from drought stress and control plants was collected for RNA sequencing. The absolute values of log2 (drought/control) ≥1 and FDR < 0.001 were used as the criteria for DEGs. The color of the box represents up (red) and down (green)-regulated (drought/normal) genes, and the value in the box is the log2 (drought/normal) of the genes in the ear leaf (drought/normal) at the 5DAP stage

Drought stress significantly reduced the photosynthesis capability of the ear leaf, which greatly contributes to the “source” for ear and kernel development as a photosynthesis factory in maize plants. In the ear leaf, GO terms related to photosynthesis were significantly enriched (Fig. 6a), with dramatically downregulated expression of photosynthesis protein and enzyme-coding genes. As shown in Fig. 6b and Additional file 2: Table S7, 21 genes involved in photosystem II, including 2 for PsbO, 4 for PsbP, 5 for PsbQ, 3 for PsbX, 2 for PsbY, 2 for Psb27 and 3 for Psb28, were downregulated in the leaf under drought stress conditions compared with the control. In the cytochrome b6/f complex, 7 genes in genes encoding PetA to D, showed significantly reduced expression levels, which was the FeS center and reduced electron transport. Twenty-four genes involved in photosystem I, including PsaA to H, PsaK, PsaL, PsaN and PsaO were downregulated. For photosynthetic electron transport, 1 gene encoding PetE, 6 genes encoding PetF and 4 genes encoding PetH/FNR were downregulated. However, 1 gene encoding PetF (GRMZM2G063126), that functions in distributing photosynthetic reducing power was dramatically induced 265-fold compared with the normal control conditions, which was the only upregulated gene in the photosynthesis systems. Eight genes in the ATPase complex were downregulated, including the genes for the gamma (1), delta (3 genes) beta (3) and b (1) subunits. Additionally, 19 genes encoding the antenna proteins were also downregulated. Plants under water deficit conditions exhibited a decline in photosynthetic rate through the control of stomatal closure by changing the turgor pressure in guard cells and activity of the PSII complex. When compare the candidate motifs in their promoter region, higher percentage of ABRE and NAC binding site were found compare to others, like MYB, ERF binding site and G-box (Fig. 6c). Figure 1 and Additional file 2: Table S7 show that the continuous decrease in net photosynthesis by drought stress in the ear leaf depends on transcription level regulation, which reduced the synthesis of some photosynthesis system proteins. This phenomenon resulted in a slow recovery for high rates of photosynthesis when drought stress was removed and led to a final yield loss compared with the normal conditions. Genes involved in photosynthesis and light harvest are listed in Additional file 2: Table S7, which implied a clear influence on the ear leaf by drought stress among the three organs.

**Fig. 7:**
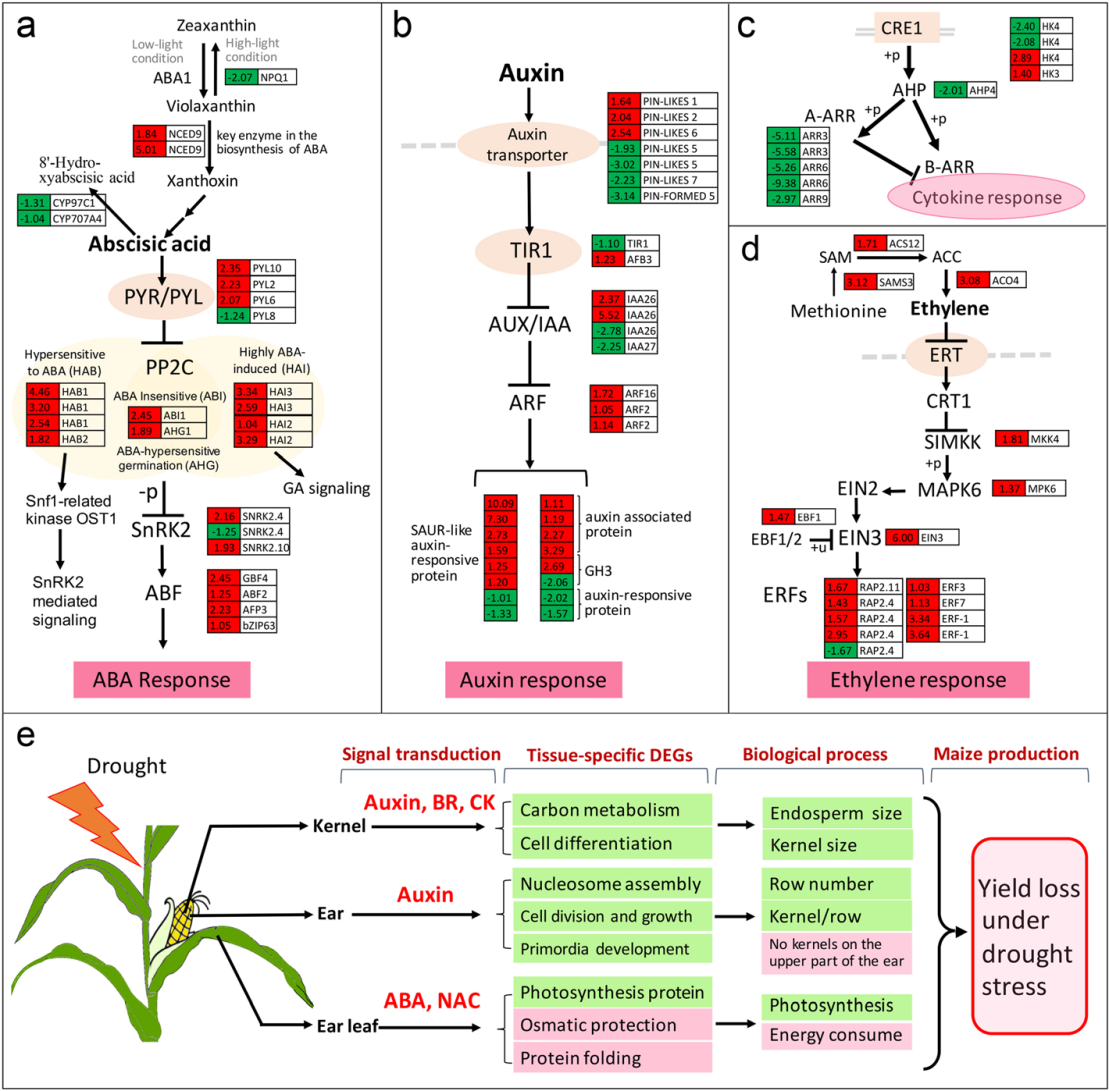
DEGs in the ear leaf (drought/normal) at the 5DAP stage involved in the plant hormone signaling pathway summary of the maize plant response drought stress. DEGs in the ear leaf (drought/normal) at the 5DAP stage involved in the ABA (**a**), auxin (**b**), cytokine (**c**) and ethylene (**d**) signaling pathway. **e** Summary of the whole plant response and organ-specific response process and their correlation to the final yield of maize under drought conditions. All the plants were grown and treated as described in Fig. 4. The ear leaf from drought stress and control plants was collected for RNA sequencing. The absolute values of log2 (drought/control) ≥1 and FDR < 0.001 were used as the criteria for DEGs. The color of the box represents up (red) and down (green)-regulated (drought/normal) genes, and the value in the box is the log2 (drought/normal) of the genes in the ear leaf (drought/normal) at the 5DAP stage

As shown in Fig. 7 and Additional file 2: Table S7, four ABA receptor genes were differentially expressed, with downregulation of the homolog of PYR8 (GRMZM2G063882, 0.42-fold) and homologs of PYR2, 6 and 10 upregulated (GRMZM2G112538, 4.70-fold, GRMZM2G112488, 4.19-fold, GRMZM2G165567, 5.09-fold) in the ear leaf. As we mentioned previously, 9 ABA-induced PP2Cs were significantly upregulated by drought stress, including four HAI homologs that function in the negative regulation of ABA signaling and positive regulation of GA signaling and five HAB homologs genes that regulate activation of the Snf1-related kinase OST1 with ABA. Three SnRK2 genes showed differential expression, with two induced (GRMZM2G110922, 4.46-fold and GRMZM2G000278, 3.80-fold) and one downregulated (GRMZM2G110908, 0.42-fold) by drought stress. Additionally, the ABF TFs (ABF2/GRMZM2G479760, 2.38-fold and GBF4/GRMZM5G858197, 5.48-fold) were upregulated and then activated the expression of ABA response genes. Ethylene signaling was active since the genes encoding SIMKK (GRMZM5G834697, 3.5-fold), MPK6 (GRMZM2G002100, 2.59-fold), EIN3 (GRMZM2G040481, 64-fold) and EBF1 (GRMZM2G171616, 2.76-fold) were upregulated. For auxin, TIR1 was downregulated (GRMZM2G135978, 0.47-fold), two homologs of IAA26 were upregulated and one IAA27 was downregulated. For auxin response genes, six SAUR genes were dramatically induced, and another two were downregulated. GA signaling was inhibited with the upregulation of GID1C (GRMZM2G173630, 3.12-fold and GRMZM2G016605, 4.03-fold) and DELLA (GAI, GRMZM2G013016, 41.61-fold) and the downregulation of two PIF3 genes at a moderate level (GRMZM2G062541, 2.25-fold and GRMZM2G387528, 0.47-fold). For the cytokine pathway, two CRE1/WOL genes (AHK4/GRMZM5G833140, 0.19-fold, and AHK4/GRMZM2G155767, 0.24-fold) were markedly downregulated and another two members were upregulated (AHK4/GRMZM2G151223, 7.43-fold and AHK3/GRMZM2G423456, 2.64-fold), these genes encode cytokine-binding receptors that transduce signals across the plasma membrane. AHP4/GRMZM2G039246, which functions as a histidine-containing phosphotransferase factor, showed 0.25-fold downregulation. In comparison to B-ARR, which did not exhibit an obvious change, five A-ARR genes were dramatically downregulated in ear leaf after drought treatment: two ARR3 homologues (GRMZM2G179827, 0.02-fold, GRMZM2G096171, 0.03-fold), two ARR6 homologues (GRMZM2G040736, 0.001-fold and GRMZM2G392101, 0.03-fold and one ARR9 homologue (GRMZM2G129954, 0.13-fold). ABA, ethylene and cytokine signaling appeared to actively regulate the response to drought stress, but GA signaling was inactive during the acclimation to drought stress.

## Discussion

### Maize adaptation to drought stress: whole-plant and organ-specific responses

When grown under drought conditions, plants attempt to adjust their adaptive mechanisms to maintain cell turgor through osmotic adjustment and water uptake and increase protoplasmic resistance to enable them to escape, avoid, or tolerate drought stress [1, 9, 16, 67]. Here, “Drought resistance” requires the communication and coordination of organ-specific and functional-specific responses. As a whole organism, plants attempt to maintain photosynthesis levels by osmotic adjustment, absorption of more water from the soil by promoting root growth, and protection of the newer organ by promoting the senescence of old leaf and reuse of water and nutrients [16, 67]. Until now, most studies have been performed at the organ-specific level, and the whole plant was largely based on theoretical models. The communication and coordination of the organ-specific responses may be largely due to the plant hormone and the carbohydrate balance between the “source” and “sink”.

Here, three organs with different biological functions and having different roles in yield loss (Fig. 1g) when maize plants were subjected to drought stress were sampled for analysis by comparative transcriptomes. Two were the important “sink” organs: the ear at the V9 stage and the kernel at 5DAP. Another was the ear leaf at 5DAP, which served as a photosynthesis factory, and drought stress reduced its net photosynthesis rate and supply capacity of “source”. Drought stress at the V9 stage led to a failure of paired spikelet differentiation on the upper part of ears and dramatically reduced the row number per ear and kernels per row. Drought stress after pollination led to delayed kernel development and abnormal endosperm cell differentiation, especially in the endosperm transfer cell layer, which decreased and finally produced small and abortive kernels (Fig. 1). Based on the transcriptome analysis, the changes in the three organs involved in the final yield of maize under drought conditions showed a good correspondence with DEGs and metabolic pathways (Fig. 7e). It was concluded that maize adaptation to drought stress was a whole plant response process, as well as an organ-specific response process. Thirteen candidate motifs were enriched in the promoters of 292 overlapped DEGs in three organs (Fig. 2f), 6 of them with the core ABRE binding site (ACGTG) and 5 with identified NAC TFs binding site. That means two candidate regulatory systems were found in all the tissues: ABA-dependent and NAC-mediated stress response pathways, and they mediated the osmotic protective substance synthesis and protein folding response under drought stress conditions. When subjected to drought stress, 1825 DEGs were detected in the ear, 3759 DEGs in the ear leaf and 6192 DEGs in the kernel, which indicated that ears were protected appropriately under drought stress conditions despite a great impact on kernel yield (Fig. 7e). The leaf, one of the most important organs for photosynthesis, usually produces more osmotic protective substances for osmotic adjustment when subjected to drought stress, and the dramatically downregulated expression of photosynthesis protein genes led to a severe reduction of the carbohydrate supply. The number of DEGs in the kernel was highest among the three organs, which was concordant with active cell division and expansion and the rapid development of this organ at this stage. Carbohydrates flow from leaves to young ears or kernels in maize. When plants suffered from drought stress, a dramatic decrease in cell division, cell differentiation and developmental processes took place in the ear and kernel because of the shortage of energy supply. It was obvious that the drought stress could disrupt or retard the developmental process of young ears and immature kernels, and organ-specific responses or the modification of metabolism and development ensued.

### Drought stress excited ABA signaling and “source” acclimation in maize ear leaf

ABA signaling plays an important role in plants in response to abiotic stresses. In this study, the ABA receptor (PYR/PYL), ABA-induced HAI and HAB, SnRK2 and ABFs were differentially expressed in ear leaf when subjected to drought stress (Additional file 2: Table S6). HAB has been reported to regulate activation of the Snf1-related kinase OST1 with ABA, and HAI is a negative regulator of ABA signaling [49]. Drought stress excited the changes in ABA-signaling pathways. However, the complex ABA signaling pathway was conserved, and species-specific differences remain to be elucidated.

In the ear leaf, dramatic downregulation of genes encoding photosynthesis proteins was observed, and dramatically decreased photosynthesis rates were observed following exposure to drought stress, including 21 genes involved in photosystem II, 7 genes in the cytochrome b6/f complex, 24 genes in photosystem I, 12 genes in photosynthetic electron transport, 8 genes in the ATPase complex and 19 genes encoding antenna proteins, all of which were downregulated in the ear leaf. Promoter analysis showed that the synergistic expression of the photosynthesis genes in response to drought stress was under the regulation of ABA- and NAC-mediated stress response pathways. The genes encoding the enzymes involved in the synthesis of sucrose, trehalose, raffinose and proline were significantly induced by drought stress, which was consistent with the soluble sugar content in the ear leaf. The changes at the transcriptional level of these genes would result in dramatically reduced photosynthesis rates under drought stress conditions. The ROS scavenging enzymes and protein folding chaperones were clearly induced by drought stress, which could facilitate scavenging of the ROS produced by the photosynthesis system and lipid metabolism of the membrane to maintain the proper conformation of the protein and the complex. Interestingly, a ferredoxin gene, *PetF,* was dramatically induced 265-fold compared with the control conditions, and overexpression of the gene in *Chlamydomonas reinhardtii* could increase the level of reduced ascorbate and diminish H_2_O_2_ levels under normal growth conditions while increasing the efficiency of ROS scavenging in chloroplasts under heat stress conditions [68]. During the acclimation of maize leaf to drought stress, the antioxidant system played a crucial role.

By comparing with the works published previous, the osmotic protective was a conserved mechanism used by maize at seedling stage [34], or flowering stage [38, 39], in the field drought stress and simulated drought stress. This was confirmed by the measurement of the physiological parameter like sugar content and leaf osmotic potential. Form the expression profile changed in the leaf response to drought stress, some common changes like the metabolic changes (carbon metabolic changes), stimulus-response were identified, however, the signaling pathways mediated the sense of drought from the environment, the amplify and transmission, and the activation of the stress response is not clear. Both forward and reverse genetic showed some evidence that ABA and transcriptional regulation played important roles in this process. However, the spatial and temporal sequence, the connection of the genes expression and their biological function are unclear. In this study, based on the gene expression changing and their promoter analysis, ABA-dependent and NAC-mediated stress response pathways seem more important in mediating the drought stress response than others.

### Drought stress inhibited the “sink” size, and plant hormone signaling played an important role in this process

The sizes of two important “sink” organs, young ears and developing kernels, were dramatically reduced when plants were subjected to drought stress, which led to a yield loss per plant with reduced row/kernel numbers and kernel size (Fig. 1 and Additional file 2: Table S1). The DEGs involved in cell division, cell growth and differentiation, organ boundary development, flower development and primordium development were significantly changed (Figs. 3, 4 and 5). Coupled with the changes in gene expression, metabolism was also greatly altered, especially carbohydrate metabolism in the kernel.

Auxin has been thought to play an important role in primordium establishment and growth. Although we did not obtain clear results for the auxin transport pathway in ears and kernels during the developmental stage, the auxin influx transporter seemed to be more important in reduced “sink” size regulation compared with PINs at the transcriptional level under drought stress conditions. The auxin influx carriers AUX1 and LAX3 are required for auxin signaling, which activates LBD16/ASL18 and LBD18/ASL20 to control lateral root development [61]. Interestingly, both the auxin-regulated NAC1 and the homolog of ATAF2, which reportedly binds to NIT2 to regulate auxin biosynthesis and to BAS1 and SOB7 to regulate BR catabolism, were found in these processes (Additional file 2: Table S3-S4). IAA7/AXR2 and IAA3/SHY2 were differentially expressed during both ear and kernel development, which indicated the existence of common developmentally regulated processes. IAA7/AXR2 and IAA3/SYH2 were identified as regulators of hypocotyl elongation and leaf shape [59, 60]. IAA7/AXR2 is a regulator of embryonic axis elongation since *axr2–1* and *axr3–1* exhibit strong insensitivity to ABA during embryonic axis elongation [59]. IAA3/SHY2 contribute to lateral root primordium development and emergence with activation of LBD16, which is an important regulator in lateral organ development [60]. In contrast to the ear, more AUX/IAA and ARFs were differentially expressed in the kernel, which demonstrated an upregulation of IAA7, IAA8, and IAA13, among others, and a downregulation of IAA16 and IAA27, among others, when subjected to drought stress. One ARF1, three ARF3, one ARF7 and one IBR were downregulated in the kernel under drought stress, while upregulated ARFs were not identified in this comparison. IAA8 has been reported to function in lateral root and floral organ development, probably by interacting with ARF6/ARF8 proteins [69]. Overexpression of IAA13 results in slower growth compared with WT [70]. A gain-of-function mutation of IAA16 confers reduced responses to auxin and ABA and impedes plant growth and fertility [71]. Others IAAs identified were largely unknown except to function as AUX/IAA proteins. ARF1 has been reported to regulate senescence and floral organ abscission in Arabidopsis. ARF3 is thought to physically interact with KANADI to form a complex for Arabidopsis organ asymmetry development [72]. ARF6 is considered the target of miR167 and to regulate female and male reproduction [73]. ARF7 is a regulator of differential growth in aerial Arabidopsis tissue [74]. It could be concluded that auxin hemostasis and signaling could play important roles in the regulation of “sink” size in maize.

In contrast to the leaf, few works by using reproductive tissue to study the drought stress, although they predetermined the final product of maize. In our previous work, DEGs with a broad range of cellular and biochemical activities were identified from the immature tassel and ear from elite inbred line DH4866 under normal and water deficit stress during meiosis stage by using Maize Oligo Array (Version 1.9) [39]. In papers from Miao et al. [36] and Danilevskaya et al. [40], RNA-seq profiling of leaves, ears, and tassels at several developmental stages (V12-R1) in response to drought stress was performed. Several GO terms were identified including the metabolism and signaling pathways for drought responses. In the work by Danilevskaya et al. [40] one difference of the treatment used from others is a high temperature fluctuation during their sampling, especially a high daily temperature was 40 °C 2 days before the first sampling, which was dual stress of heat and drought stress especially for B73 that is a typical temperate line. Comparative analysis between these published results and ours concluded that drought caused massive changes during developmental processes in ear and leaf tissues, and water balance was maintained by co-expression of the genes involved in osmotic adjustments and transporter proteins, drought-induced gene expression changes also occurred in a tissue-dependent or developmental-stage manner, although some DEGs involved in ABA-dependent signaling, and phosphoprotein cascades, reduced photosynthesis, etc. showed inconsistency in the different maize lines. The signaling and transcriptional regulators were not elucidated enough for the precision and the annotation of the maize genome at this time. Based on Akshay et al., compared to leaf meristem tissue, the metabolic processes were much more affected in the fertilized ovary including the carbon metabolism. These were also found in our result by using the 5DAP kernels. In morphological, physiological and comparative transcriptomics analysis for understanding the different features of “sink” or “source” organs and the effects on kernel yield under drought stress conditions, the ABA-, NAC-mediate signaling pathway, osmotic protective substance synthesis and protein folding response were identified as common drought stress response in the leaves, ear primordia and young kernels, while tissue-specific drought stress responses and the regulators were also identified, they were highly correlated with growth, physiological adaptation and yield loss under drought stress. For ears, drought stress inhibited ear elongation, led to the abnormal differentiation of the paired spikelet, and auxin signaling involved in the regulation of cell division and growth and primordium development changes. In the kernels, reduced kernel size caused by drought stress appeared with the obvious differences of auxin, BR and cytokine signaling transduction, the modification in carbohydrate metabolism, cell differentiation and growth retardation. Transcriptomic changes caused by drought were highly correlated with developmental and physiological adaptation, which was closely related to the final yield of maize, and a sketch of tissue-specific responses to drought stress in maize was clarified the new findings of this study.

### Improving maize yield under drought stress conditions: dream and realization

Better crop performance in drought environments is imperative for food security in the face of climate change. Thus, strategies to improve drought stress tolerance will be a hotspot among all plant breeders. Some issues must be considered to provide a proper evaluation. First, measurement and selection of the drought-resistant period is necessary, for plant growth and development is affected by stress in a tissue- or organ-specific manner. Although we can obtain a wealth of knowledge using maize seedling materials for drought treatment, they lack key roles for the improvement of breeding. Drought stress around the time of flowering had a greater influence on the development of reproductive organs and the final yield of maize. Second, common and organ-specific mechanism can be used for breeding. One of the most productive ways to tackle the agricultural challenge of drought is through cross-fertilization between lines with drought tolerance in different development phases and good agronomy performance. Such techniques should be used with caution for organ-specific mechanisms for improved responses to drought stress. When an organ-specific promoter is used to drive a drought response gene in transgenic plants, we could improve drought resistance with the least yield penalty. Third, the balance among carbohydrates between “source” and “sink”, between osmotic adjustment and cell division and growth are important for maize breeding. Carbohydrates serve not only as a necessary major nutrient for cell division and growth but also as a signaling molecule in the plant body. The maintenance of maize yield stability under drought stress conditions requires further study to understand responses at posttranscriptional and protein levels.

## Conclusions

Drought is a serious causal factor of reduced crop yields than any other abiotic stresses. As one of the most widely distributed crops, maize plants frequently suffer from drought stress, which causes great losses in the final kernel yield. In this study, three organs of maize plants, “source” organ the ear leaf, “sink” organs young ears at the V9 stage and kernels at the 5DAP stage were used for comparative transcriptomics analysis to reveal the differences of drought stress response in these organs and help to understand the mechanism of yield lost induced by drought stress. It was concluded drought-induced gene expression changes occurred in a tissue-dependent manner. For immature ears, drought stress inhibited ear primordium elongation and led to abnormal differentiation of the paired spikelet, and aroused the changes of auxin signaling regulation involved in cell division, cell growth and primordium development. For kernels, reduced kernel size caused by drought stress was significant, and plant hormones, such as auxin, BR, and GA, and cytokine signaling pathways changed greatly in the drought stress response, which affected carbohydrate metabolism, cell differentiation and growth retardation. For the ear leaf, as “source” organ, drought stress excited ABA signaling and “source” acclimation, the expression of many genes encoding photosynthesis proteins were dramatically reduced. Retarded growth correlated with the down-regulation of DNA replication and cell-cycle genes and the up-regulation of ABA signaling, and common drought stress responses were also existing in the three organs to mitigate the effects of drought. These findings provide an organ-specific response network in maize exposed to drought stress around the flowering stage and aid in understanding the mechanism of the drought stress response to reduce kernel yield in maize and further guide maize breeding.

## Methods

### Plant materials

Plant materials used in this study were from the maize inbred line B104. B104 is an inbred line derived from an Iowa Stiff Stalk Synthetic population (BS13(S)C5). B104 has consistently high yield performance in crosses with Mo 17 and B97 and shares approximately 93% genetic similarity with B73 [75].

### Drought stress treatment of plants and yield determination

Field experiments for the drought were performed in the maize growing season. Two different drought stress treatments (5 days drought stress at the V9 stage and 5DAP stage) and a normal control were performed (Fig. 1a). The trial plots (4 rows/plot, 10 plants/row, 66,700 plants/ha) were arranged in a randomized complete block design with four replications. A 30 cm impermeable zone between plots (30-cm-deep embedded plastic sheeting) was set up. All the plants were grown under natural conditions (200–1600 mmolm^− 2^ s^− 1^ at noontime, 20–35 °C, normal nutrients, well-watered with soil water content (SWC) ≥19.5%) until the designated stages. Soil water content was monitored by using a Soil Moisture Content Meter (TZS, TOP instrument, China). For drought stress at the V9 stage (when the 10th leaf appeared), plants were subjected to drought stress treatment for 5 days by maintaining 14.0–15.0% SWC (when SWC decreased to 15%, 1 day was recorded), and then they were rehydrated for recovery growth. The upper ear (at the spike differentiation stage) from drought stress and control plants was collected for morphological analysis and RNA sequencing. For drought stress at the 5DAP stage (when the silk appeared), plants were grown under natural conditions until the pollination stage, and then they were subjected to drought stress treatment by maintaining 14.0–15.0% SWC, followed by the restoration of watering. Ear leaf and kernels from the plants at 5DAP that had suffered 5 days of drought treatment and the control materials were sampled for morphological observation and RNA sequencing. The remaining plants grew to maturity under suitable conditions, and then the ears were harvested to determine the kernel yield, ear length and number of kernel rows. For the control group, plants were grown under their normal natural conditions. At least three biological repeats were sampled for a treatment, and each repeat contained organs from 4 plants.

### RNA extraction for the samples used for RNA sequencing analysis and real-time RT-PCR

Total RNA was isolated as described by McCarty because of the high sugar content in the ear, kernel and ear leaf organs [76]. Briefly, 0.5 g fresh weight samples were ground up using a baked mortar and pestle with liquid nitrogen. Then, the powder was transferred into a 7-ml tube with 2 ml of extraction buffer (100 mM Tris-HCl, 200 mM NaCl, 20 mM EDTA, 1% (m/v) sodium laurate, 5 mM DTT) and vortexed well. The samples were then extracted using phenol: chloroform: isoamyl alcohol (24:24:1, v:v:v) and chloroform. After LiCl and ethanol precipitations, the RNA quality was examined by the UV absorbance spectra, gel electrophoresis and Aligent 2000. The protocol can be used to obtain high-integrity, translatable mRNA in maize tissues. Real-time RT-PCR of the candidate genes and data analysis were performed as described by Zhang et al. [77] and primers used were list in Additional file 2: Table S8.

### RNA sequencing library construction and data analysis

Tag preparation, DNA purification and Illumina sequencing, and primary bioinformatics analysis were performed by BGI Tech Solutions Co., Ltd. (Shenzhen, China) according to the standard procedure (Additional file 1: Figure S3). Briefly, after treated with DNase I, the mRNA is enriched by using the oligo (dT) magnetic beads, and then fragmented into short fragments. Then cDNA was synthesized, purified with magnetic beads and 3′-end single nucleotide A (adenine) was added. Finally, sequencing adaptors are ligated to the fragments and amplified by PCR. Agilent 2100 Bioanaylzer and ABI StepOnePlus Real-Time PCR System are used to qualify and quantify (QC) of the sample library. Primary sequencing data (raw read) were produced by Illumina HiSeq™ 2000. After QC, raw reads are filtered into clean reads which will be aligned to the reference sequences. The alignment data is utilized to calculate distribution of reads on reference genes and mapping ratio. Then the downstream analysis (PCA/correlation/screening DEGs (FDR ≤ 0.001 and the absolute value of Log2Ratio ≥ 1 as the threshold to judge the significance of gene expression difference)) were performed (GEO Submission (GSE132113)). Further, case special deep analysis based on DEGs including GO enrichment analysis, pathway enrichment analysis, cluster analysis, promoter motif analysis and were done by our group. GO analysis was performed using AgriGO (http://bioinfo.cau.edu.cn/agriGO/). Pathway analysis was based on KEGG (http://www.genome.jp/kegg/). The upstream regions of the genes for common sequence motifs using PromZea (http://128.196.172.219/index.html).

### Physiological index determination

The net photosynthesis rate was measured in the ear leaf of plants using a portable infrared gas analyzer-based photosynthesis system (LI-6400, Li-Cor Inc., Lincoln, NE, USA). The fresh weights (FW), turgid weights (TW, soaked in deionized water at 4 °C overnight) and dry weight (DW, oven-drying of leaf samples at 70 °C for 72 h) of leaves were recorded after excising them from maize plants. The relative water content (RWC) was calculated as follows: RWC (%) = (FW − DW)/(TW − DW) × 100%. The solute potential (ψs) was measured with a cryoscopic osmometer (Model 210, Fiske, Norwood, MA, USA) and calculated using the following formula: ψs = −moles of solute (RK), where R = 0.008314 and K = 298 °C [78]. Total soluble sugars of leaves (approximately 0.1 g) were extracted in boiling water for 30 min and determined by anthrone reagent using glucose as the standard [79].

### Scanning electron microscopic observation of ears

The ears of control and drought stress treatment plants were observed using scanning electron microscopy (SEM). After fixation with glutaraldehyde and dehydration with ethyl alcohol, pieces of the ears were prepared by removing the top part of the ear using a razor blade. The samples were then loaded on SEM specimen stubs and sputter-coated with gold (Cressington Sputter Coater 108, Cressington Scientific Instruments Inc., Watford, Hertfordshire, UK). The specimens were scanned and photographed using an FEI Quanta FEG 250 environmental scanning electron microscope at 5 kV under a high vacuum.

### Paraffin section observation of kernels

The anatomical morphology of the kernels was observed using paraffin sections. The kernels were collected from the ears at 5DAP and then fixed in 50% FAA (containing 50% ethanol and 4% formaldehyde) for 24 h. The samples were dehydrated in an ethanol gradient series from 50 to 100%. The samples were then cleared using xylene and embedded in paraffin. The samples were sectioned at a thickness of 12 μm, stained with toluidine blueO and observed with an Olympus BX51 microscope.

## Additional files

Additional file 1 : **Figure S1** Ears at the V9 stage and kernels at the 5DAP stage under normal and drought stress conditions. **Figure S2** Validation of DEG identified in RNAseq by using real-time RT-PCR. **Figure S3** The flow-chart of the RNA-sequencing experimental process and bioinformatics analysis pipeline from BGI (BGI Genomics). **Table S1** Agronomic traits of maize plants grown under control or drought stress conditions at different developmental stage in the fields. **Table S2** The overall of the RNAseq used in this paper. (DOCX 11396 kb)

Additional file 2 **Table S3** Key differentially expressed genes involved in the ear at the V9 stage and the kernel and leaf at the 5DAP stage subjected to drought stress. **Table S4** Key differentially expressed genes involved in ears at the V9 stage subjected to drought stress. **Table S5** Key differentially expressed genes involved in carbohydrate metabolism and plant hormone signaling in kernels at the 5DAP stage subjected to drought stress. **Table S6** Key differentially expressed genes involved in the kernel at the 5DAP stage subject to drought stress. **Table S7** Key differentially expressed genes involved in the ear leaf at the 5DAP stage subjected to drought stress. **Table S8** Primer sequence used in this paper. (XLSX 139 kb)

## Data Availability

All data and materials generated or analyzed during this study are included in this article or are available from the corresponding author on reasonable request. The expression data reported are available in the NCBI Gene Expression Omnibus (GEO) database under the GSE series accession number GSE132113.

## Abbreviations

ABA: Abscisic acid
ABRE: ABA-responsive element
DAP: Days after pollination
DEGs: Differentially expressed genes
GO: Gene ontology
ROS: Reactive oxygen species
RPKM: Reads Per Kilobase Million
RWC: Relative water content
SEM: Scanning electron microscopy
SWC: Soil water content
TF: Transcription factor
