# Supplementary material for: Effects of maize organ-specific drought stress response on yields from transcriptome analysis
Source: BMC Plant Biol. 2019 Aug 1;19:335. doi: 10.1186/s12870-019-1941-5 (PMC6676540; doi:10.1186/s12870-019-1941-5)
Supplement: Supplementary file 1 — : Figure S1 Ears at the V9 stage and kernels at the 5DAP stage under normal and drought stress conditions. Figure S2 Validation of DEG identified in RNAseq by using real-time RT-PCR. Figure S3 The flow-chart of the RNA-sequencing experimental process and bioinformatics analysis pipeline from BGI (BGI Genomics). Table S1 Agronomic traits of maize plants grown under control or drought stress conditions at different developmental stage in the fields. Table S2 The overall of the RNAseq used in this paper. (DOCX 11396 kb) [file 12870_2019_1941_MOESM1_ESM.docx]

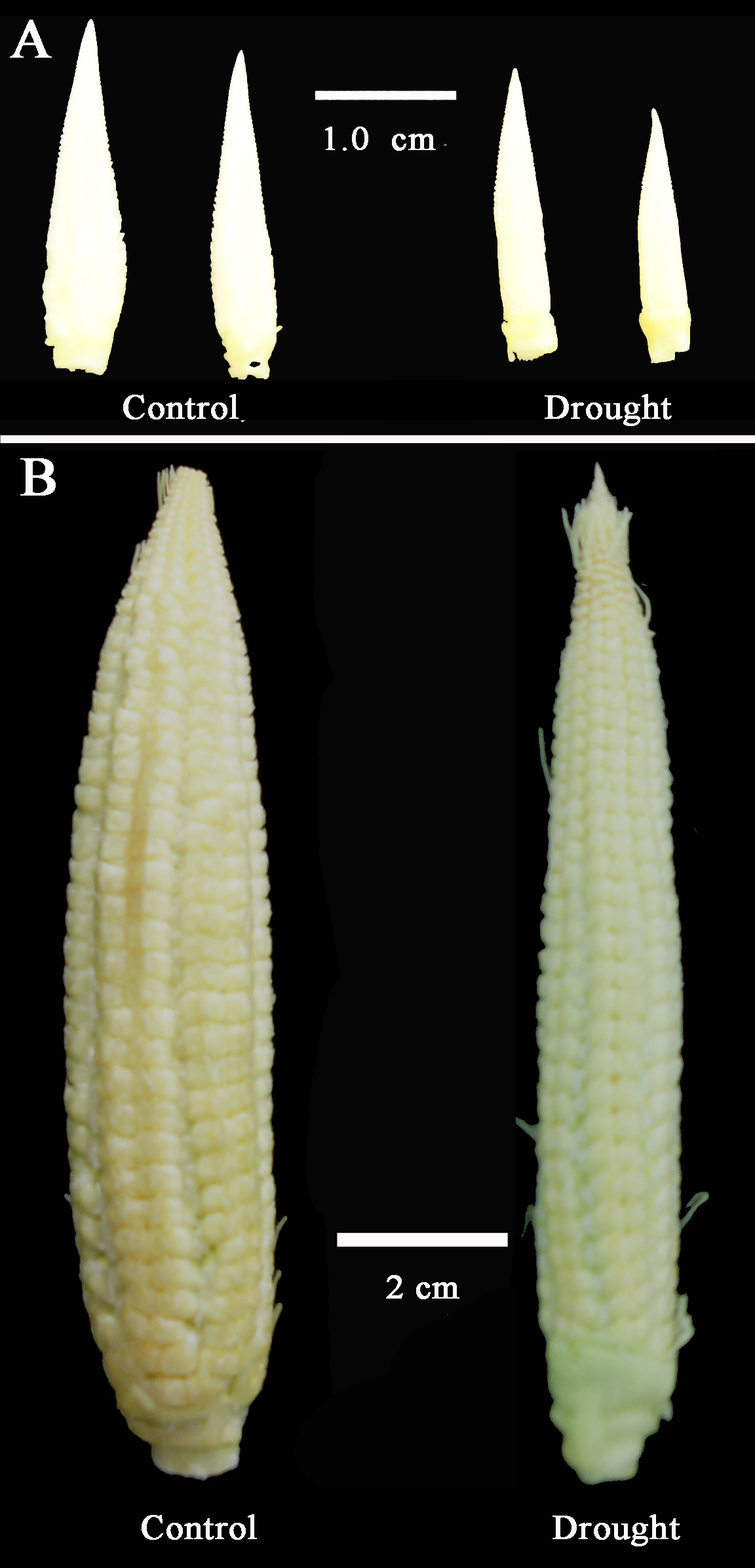


Fig. S1 Ears at the V9 stage and kernels at the 5DAP stage under normal and drought stress conditions

A. The ears at the V9 stage under normal and drought stress conditions; B. the kernels at the 5DAP stage under normal and drought stress conditions.

Ears were form field experiments. All the plants were grown under natural conditions, normal nutrients, well-watered soil until the designated stages. Two different drought stress treatments (5 days drought stress at the V9 stage and 5DAP stage) and a normal control were performed. The upper ear from drought stress and control plants was collected for morphological analysis and RNA sequencing.


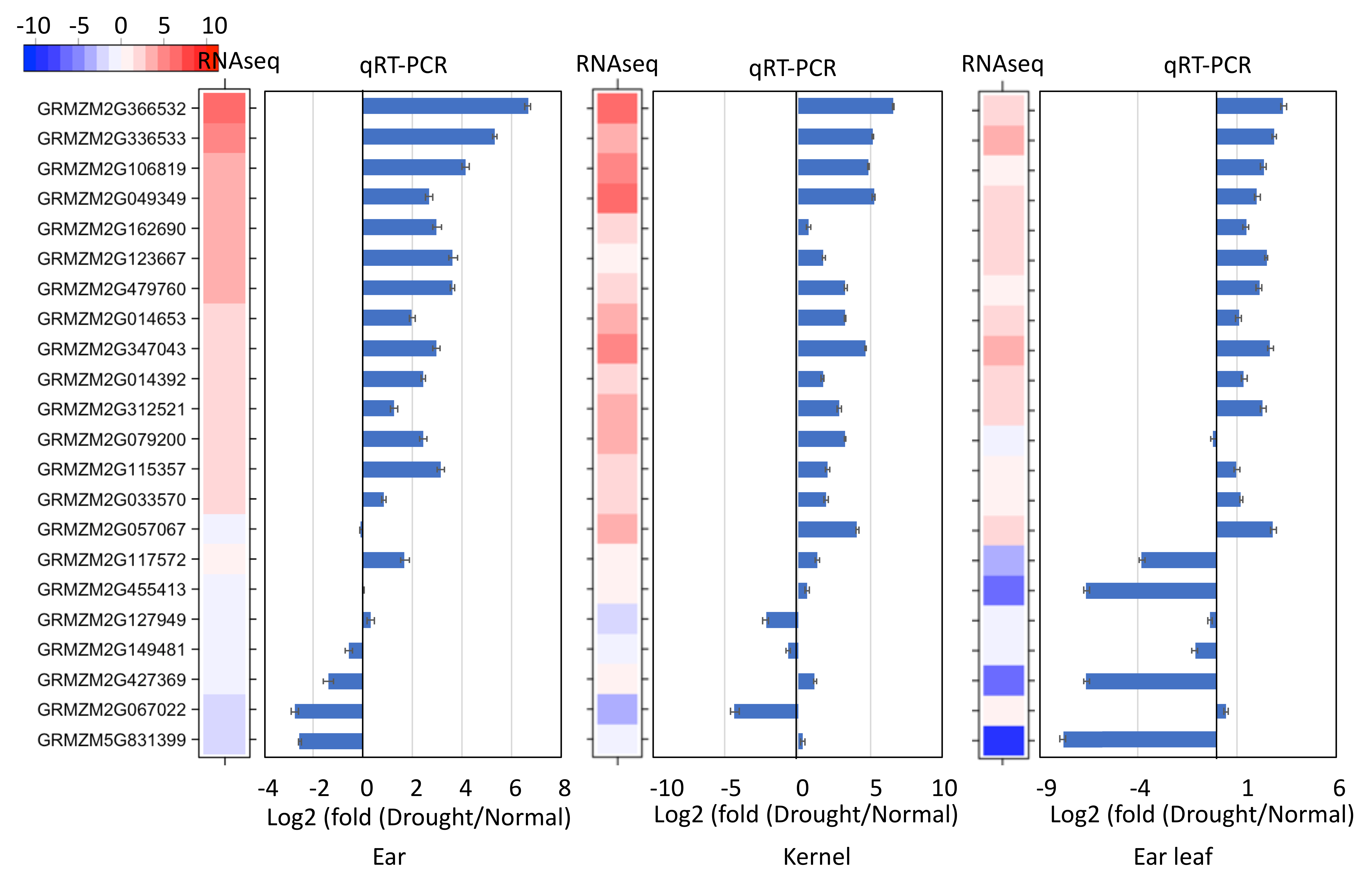


Fig. S2 Validation of DEG identified in RNAseq by using real-time RT-PCR­­

The left heatmap showed the log2 fold changes of the DEGs identified in RNAseq. And the bar graph were the result by using real-time RT-PCR. Log2 values (Drought/Normal control) were used to generate the plot. Expression levels of genes were analyzed by real-time RT-PCR, fold changes in transcripts were calculated by the 2-ΔΔCt method with maize *actin1* as an internal control and the values of normal conditions was set as 1 fold. Samples used were same as described in Figure2.

Fig. S3 The flow-chart of the RNA-sequencing experimental process and bioinformatics analysis pipeline from BGI (BGI Genomics)

Flow-chart showed the steps for the experimental and bioinformatics analysis pipeline. The total RNA samples are first treated with DNase I to degrade any possible DNA contamination. Then the mRNA is enriched by using the oligo(dT) magnetic beads. Mixed with the fragmentation buffer, the mRNA is fragmented into short fragments. Then the first strand of cDNA is synthesized by using random hexamer-primer. Buffer, dNTPs, RNase H and DNA polymerase I are added to synthesize the second strand. The double strand cDNA is purified with magnetic beads. End reparation and 3’-end single nucleotide A (adenine) addition is then performed. Finally, sequencing adaptors are ligated to the fragments. The fragments are enriched by PCR amplification. During the quality control (QC) step, Agilent 2100 Bioanaylzer and ABI StepOnePlus Real-Time PCR System are used to qualify and quantify of the sample library. The library products are ready for sequencing via Illumina HiSeq^TM^ 2000.

Primary sequencing data that produced by Illumina HiSeq^TM^ 2000, called as raw reads, is subjected to QC that determine if a re-sequencing step is needed. After QC, raw reads are filtered into clean reads which will be aligned to the reference sequences. QC of alignment is performed to determine if re-sequencing is needed. The alignment data is utilized to calculate distribution of reads on reference genes and mapping ratio. If alignment result passes QC, we will proceed with downstream analysis including gene expression and deep analysis based on gene expression (PCA/correlation/screening differentially expressed genes and so on). Further, we also can perform deep analysis based on DEGs (We use 'FDR ≤ 0.001 and the absolute value of Log2Ratio ≥ 1' as the threshold to judge the significance of gene expression difference), including Gene Ontology (GO) enrichment analysis, pathway enrichment analysis, cluster analysis, protein-protein interaction network analysis and finding transcription factor.

Table S1 Agronomic traits of maize plants grown under control or drought stress conditions at different developmental stage in the fields

| Conditions | Ear length (cm) | Ear width (cm) | Weight per ear (g) | Ear row number | Grain numbers per row | Hundred- grain dry weight (g) | Yield (kg/plot) |
| --- | --- | --- | --- | --- | --- | --- | --- |
| Control | 12.36±0.33 | 3.67±0.25 | 59.75±1.51 | 14.00±0.00 | 22.33±0.94 | 20.33±0.13 | 2.31±0.03 |
| Drought stress at the V9 stage | 10.01±0.45** | 3.46±0.09 | 39.23±2.92** | 12.00±0.00** | 17.33±0.94** | 22.56±0.22* | 1.57±0.06** |
| Drought stress at the 5DAP stage | 11.77±0.31 | 3.21±0.08* | 50.38±0.32** | 14.00±0.00 | 21.00±1.63 | 17.38±0.41** | 2.01±0.05** |

Data were form field experiments. All the plants were grown under natural conditions, normal nutrients, well-watered soil until the designated stages. Two different drought stress treatments (5 days drought stress at the V9 stage and 5DAP stage) and a normal control were performed. After re-watering, the plants were grown to maturity under suitable conditions, and then the agronomic traits were determined. Three biological repeats were sampled for a treatment, and each repeat contained organs from 4 plants. Values are the means of the replicates ± sd. * was statistical significance with P<0.05, and ** was statistical significance with P<0.01 by using a t-test.

Table S2 The overall of the RNAseq used in this paper
